# Dietary Habits and Lifestyle, Including Cardiovascular Risk among Vegetarians and Omnivores during the COVID-19 Pandemic in the Polish Population

**DOI:** 10.3390/nu15020442

**Published:** 2023-01-14

**Authors:** Izabela Kwiatkowska, Jakub Olszak, Piotr Formanowicz, Dorota Formanowicz

**Affiliations:** 1 Department of Medical Chemistry and Laboratory Medicine, Poznan University of Medical Sciences, 61-701 Poznan, Poland; 2 Institute of Computing Science, Poznan University of Technology, 60-965 Poznan, Poland

**Keywords:** COVID-19, lockdown, vegetarian diet, vegan diet, cardiovascular risk, diet quality, dietary habits, lifestyle, behavioral factors

## Abstract

**Background:** This study assessed how two food groups—omnivores (OMN) and vegetarians (VEGE)—differ in lifestyle changes, including dietary habits during the COVID-19 pandemic. **Materials:** A total of 861 persons participated in the survey and were divided into two groups: persons following a mixed diet (*n* = 489) and vegetarians, including vegans (*n* = 372). The mean age shows no significant differences. **Methods:** An online survey was conducted on the Polish population during the COVID-19 pandemic. Data was collected using social media; the survey was intended for adults and included separate sheets for different diets (OMN vs. VEGE). **Results:** The results in both groups were similar regarding the burden of premature diseases. Most respondents (~90%) did not indicate cardiovascular disease abnormalities. In the OMN group, overweight and obesity occurred more often, and the OMN group also showed a higher percentage of people reporting weight gain (OMN 42.7% vs. VEGE 35.9%). The results disclosed the VEGE group significantly more frequently chose products, i.e., vegetables (*p* = 0.029), legumes (*p* < 0.001), and dairy products or their plant substitutes (*p* = 0.002), compared to the OMN group. **Conclusions:** The VEGE group revealed the most regularities in dietary habits during the pandemic.

## 1. Introduction

In 2020, due to the emergence of the novel coronavirus disease (COVID-19), the World Health Organization (WHO) announced a global pandemic [1]. There were numerous changes concerning the everyday functioning of society. Government recommendations introduced numerous restrictions, including new rules for the functioning of public and commercial institutions, new entities of education systems (e.g., remote work, closing kindergartens, schools, and universities), the need to stay at home (lockdown), and observing the rules of social isolation and behavior in the broad sense of distance [2]. The new rules were intended to protect the health of society. Still, through their radical form, they could significantly affect many other areas related to health, including the components of an individual’s lifestyle, e.g., eating habits and level of physical activity [3,4,5,6,7].

Stress caused by the necessity of lockdown could cause many behavioral changes; the described possible impact on the human psyche considers such consequences as depressed mood, irritability, insomnia, anger, exhaustion, and emotional disorders, including depressive symptoms [8,9,10,11,12,13]. Such events, where the whole world is “quarantined” (worldwide lockdown) due to a new, unknown, deadly disease also have an impact on eating behaviors, where mood or emotional state plays a huge role and has the potential to change them [14,15,16,17,18]. Changes in eating behavior may include an increase or decrease in the number of meals, snacks, ready-made products, and stimulants consumed, which may have consequences in the change in body weight. There may also be beneficial consequences associated with the necessity of nationwide quarantine, like preparing meals at home, changing food choices to healthier, unprocessed products, or the possibility of spending more time on physical activity [3,5,19,20,21,22,23,24,25]. The results of numerous global studies show various effects of the COVID-19 pandemic on changing eating habits and physical activity [6,26,27,28,29,30,31,32,33,34].

Behavioral factors significantly impact health. WHO indicates that lifestyle components, including excess body weight, stimulants, and low physical activity, are significant risk factors for premature death due to non-communicable diseases, including cardiovascular diseases (CVD) [35,36,37]. World reports/recommendations attach great importance to a low-quality diet [37,38,39]. Cardiovascular risk factors include modifiable and non-modifiable factors. According to the latest recommendations of the European Society of Cardiology from 2021, the main risk factors influenced by humans were dyslipidemia, hypertension, smoking, diabetes, and obesity [40]. Excessive consumption of products rich in sugar, poor-quality fats (saturated and trans fatty acids), and salt is a growing trend on a global scale and is responsible for 1/3 of diseases. In this connection, the need for action against unhealthy nutrition and raising awareness of the importance of lifestyle for health is emphasized [41]. The EAT–Lancet Commission Summary Report (2019) stressed that the aspects related to food are one of the leading health and environmental challenges; to achieve a healthy diet, it was recommended to increase the consumption of plant products and significantly reduce the consumption of food of animal origin [39].

In various countries, different populations may have other eating habits that affect health; one example is people giving up meat, commonly called vegetarians. This lifestyle has been advocated in Hinduism and Buddhism as a way of life since their conception. The history of vegetarian nutrition started in the sixth century BC; the first vegetarian society was established in England in 1847, then the International Vegetarian Society was founded in 1908, and the first Vegan Society began in 1944 [42,43,44]. Recently, these diets have become better known and recognized by global institutions and organizations, and according to them, only a properly balanced diet can bring health benefits [45,46,47,48].

Vegetarian diets are credited as having many positive effects regarding preventive action and improving health (e.g., most often, people following this diet are characterized by a lower ratio of CVD, cancer or diabetes incidence, obesity, and hypertension) and environmental impact [49,50,51,52,53,54,55]. There are also promising results from studies on people adhering to these diets regarding better results of modifiable risk factors (overweight and obesity, lower concentration of total cholesterol, low-density lipoprotein cholesterol (LDL-C), and apolipoprotein B) for cardiovascular disease and results showing their possible therapeutic effect [56,57,58,59]. If the diet is not adequately balanced, there is a risk of deficiencies, which will negate the beneficial effects of the diet; hence, there is continual controversy around these diets due to possible deficiencies [45,46].

Vegetarian diets are becoming increasingly popular in Western countries [60,61,62,63]. It is a diet that includes many variations that limit animal products, from most animal products to non-animal products (semi-vegetarians, pescatarians, lacto-vegetarians, lacto-ovo vegetarians, ovo-vegetarians, vegans) [60,64,65,66,67]. The main reasons for using a vegetarian diet include ethical concerns and health benefits, environmental, religious, and financial aspects, or altered perception of food (especially taste, view, or smell) [68,69,70,71,72]. The reasons that guide the maintenance of the chosen diet may translate into the quality of the diet used, e.g., through the choices made concerning everyday nutrition. Researchers report that vegetarians are mainly motivated by health reasons [66,73,74,75]; in addition, the higher quality of the diet used by such people has been shown compared to other vegetarians motivated by different aspects [73].

The emergence of the pandemic caused difficulties in the work of health services, and the number of patients constantly increased. Lifestyle, including diet, significantly affects health with regards to preventing or causing bad health, and also has a long-term effect on health. Perhaps groups that follow a particular diet, e.g., resigning from meat because of different dietary motivations and in connection with ideology because of a higher sensitivity, show more correct behaviors and the possibility of maintaining it even during the pandemic. However, due to high sensitivity, they may demonstrate an increased desire for nutritional compensation (e.g., manifested by increased snacking, reaching for unhealthy snacks and drinks) related to challenging experiences and excessive emotions.

Most of the analyzed studies from a systematic review (2021) by Khan et al., from different countries of the world (taking into account changes in eating habits) mainly show an increase in food intake during the pandemic [76]. None of these reviewed studies included people using different types of diets. The cited review showed positive and negative changes in the responders’ lifestyle quality (including eating habits) during the pandemic [76]. A systematic review by Bennett et al., based on twenty-three articles, showed a reduction in purchasing and consuming fresh produce, including fruit and vegetables (nine studies) and an increase in comfort foods (six studies) [3]. This study also revealed a rise in alcohol consumption (two studies), decreased physical activity (seven studies), and weight gain (eight studies) [3]. There is a smaller number of studies that observed positive effects: changes in increasing the consumption of fresh produce (five studies), increased time spent on preparing home-cooking meals (three studies), a reduction in comfort foods (two studies), and also a decrease in alcohol consumption (three studies) [3].

This study aimed to check how, in the Polish population, two food groups—omnivores (OMN) and vegetarians (VEGE)—differ regarding the quality of life changes, including dietary habits during the pandemic. In addition, due to the numerous controversies surrounding vegetarian diets, we wanted to check how these people differ in their cardiovascular risk and health status (declared individually) compared to the group of omnivores.

## 2. Materials and Methods

An online survey was conducted on the Polish population during the COVID-19 pandemic. The survey was available for the duration of the 2nd, 3rd, and 4th waves of the pandemic in Poland (December 2020–November 2021). Data was collected using social media, the survey was intended for adults, and it was completed individually. To recruit people on vegetarian diets, including vegan diets, we added advertisements in closed groups for people following such a diet in Poland (“Vegetarians and Vegans” with more than 24,000 members and “Vegans Poland” with 59,000 members). The prepared questionnaire included questions about self-declaration, whether the participant follows a vegetarian or vegan diet, and for what period of time (see Questionnaire S1).

Separate sheets of the questionnaire were created for people who adhered to different diets: traditional/mixed (OMN) (including meat consumption) and vegetarian, including vegan (VEGE) (eliminating meat consumption), (see Questionnaire S1 and S2). The volunteers took part in a survey released online, mainly through social media. Anyone could complete the survey if classified under the required restrictions regarding age (>18 years old) or, in the case of the VEGE group, following a non-meat diet for at least six months. The average time required to complete the survey was approximately 10 min. A total of 861 participants completed the survey (which was the final number of respondents for the analyses). Most of the questions were obligatory, and for the remaining questions (optional or multiple choice), if the answer was not given, it was not taken in the final question summary (the number “n total” was then reduced). From the respondents’ data, the exclusion of “double-sent” sheets was made (which were removed as soon as they appeared by the person managing the answers received, such a situation occurred in eleven examples). The study was carried out following the Declaration of Helsinki of the World Medical Association and approved by the Bioethics Committee at Poznan University of Medical Sciences (No. 237/20/2020).

Two main groups were distinguished in the study: people following a mixed diet (OMN) (*n* = 489) and people resigning from meat consumption—vegetarians, including vegans (VEGE) (*n* = 372). The survey was aimed at adults; the average age of respondents indicates that the leading target group was young adults (mainly women) with higher than secondary education residing in the city.

### 2.1. Diet and Lifestyle Behaviours Questionnaire

Before the questionnaire was sent out, a pilot study was conducted, which showed a need for possible improvements. Medical experts were then asked to comment on and possibly validate the questionnaire, and all suggestions were taken into account. Each expert approved the validity of using the proprietary questionnaire.

The survey consisted of 4 sections, taking into account the following:

- Primary data of respondents/socio-demographic data (number of questions = 9 for OMN, 11 for VEGE, the difference is due to additional questions about a vegetarian diet).
- Information on cardiovascular risk factors (number of questions = 16).
- Changes caused by the epidemic situation, taking into account the quality of life (number of questions = 13).
- Changes in eating habits during the pandemic (number of questions = 19 for OMN and 18 for VEGE (the difference is due to an additional question about meat consumption in the OMN group)).

Some questions allowed assigning numerical values to individual answers, e.g., possible responses ranged from “never/almost never” to “daily.” By assigning each possible answer a number, e.g., from one to five, the mean frequency of consumption for each product in the study groups was calculated.

### 2.2. Statistical Analysis

Qualitative variables are described by the number (*n*) and frequency (%), and the measurable variables are characterized by the arithmetic mean (Mean) and standard deviation (SD) values.

Due to the nature of the variables (measurable variables described on an ordinal scale and the lack of normal distribution of quantitative variables), non-parametric tests were used for statistical analyses. Statistical analysis of the obtained results was performed using the statistical programs Jamovi [77] and SPSS [78] (for multiple-response data analysis). The Shapiro–Wilk and Levene’s homogeneity tests were performed to check normal distribution and equal variances of age and BMI accordingly. Due to the violations of both, the analysis was based on a non-parametric method. The Mann–Whitney U test was used to compare both study groups for ordinal variables. For nominal variables, the chi-square (χ^2^) test was used. All *p* values were two-sided and statistical significance was set at *p* < 0.05.

For the evaluation of questions regarding blood pressure, smoking, or alcohol consumption for calculating *p* values, the number was assigned for given answers. Assigned numerical values started from 0 (for meaningless/negative response) and increased as the intensity of the trait/value of the response increased. Regarding blood pressure characteristics, the answers “I don’t know” and “do not measure” were not included in the significance test.

## 3. Results

### 3.1. Baseline Characteristics

Eight hundred sixty-one people participated in the survey; most respondents were women (Table 1). The mean age of the respondents did not show any statistically significant differences; in the OMN group, it was 27.6 years, while in the VEGE group, it was 28.2 years (no significant differences, *p* = 0.744) (Table 1). Approximately 85% of the sample had a university or university college level of education in both groups (which showed significant differences, *p* = 0.025). The vast majority of respondents came from a city (*p* = 0.001).

The average duration of adherence to the vegetarian diet among the VEGE group was 5.9 years (this information was not required in the OMN group).

### 3.2. Cardiovascular Risk Factors of the Respondents

Among the studied groups, the burden of premature diseases related to lipid and carbohydrate metabolism was compared by asking whether these abnormalities occur/occurred among first-degree relatives (Table 2).

Both groups indicated an approximately 50% genetic load of CVD and the presence of abnormalities in the lipid profile. Moreover, abnormal glucose values and related conditions in relatives of the subjects were found in almost 35% of cases. However, these results were statistically insignificant in both groups.

It was checked whether the respondents showed any illnesses (Table 3).

It has been shown that incorrect carbohydrate metabolism values are more common in people on a mixed diet by 6.4 p.p. (percentage points) and showed a significant difference between the studied groups. Most of the respondents did not indicate any abnormalities characteristic for CVD (approx. 90%). Abnormal lipid profile values were rare; they concerned approximately 11% of respondents in both groups, mainly related to total cholesterol.

When comparing the pressure value in the subjects (Table 4), most of the respondents in both groups had average normal blood pressure levels (the correct answers were <120/60 and 140/80–120/60); the results of both groups (and when including gender) did not differ significantly. A large percentage of respondents in both groups did not know their blood pressure or did not measure it (24.9% OMN vs. 30.1% VEGE).

The respondents’ answers show that most people in both groups did not smoke (Table 4). The OMN group was 7.6 p.p. more likely to choose the answer “no” regarding smoking. The VEGE group showed significantly more tobacco smoking (*p* = 0.003) than the OMN group.

Similar alcohol consumption was demonstrated in both groups; the most common answer was a response indicating low alcohol consumption, and the results in the groups did not differ significantly (Table 4).

The VEGE group was characterized by a significantly lower mean body weight of 6 kg and a BMI value of 21.8 kg/m^2^. In contrast, the omnivore group’s BMI value was 23.45 kg/m^2^ (Table 5). Overweight and obesity occurred more frequently among people on a mixed diet than among the VEGE group; almost 27% of people from the OMN group were overweight or obese, while this percentage in the second group was 16.1%. More than 13.2% of underweight cases were found in the VEGE group. The division of the respondents according to the BMI classification is presented in Table 6.

The frequency of consumption of products that are a source of fat (among the macronutrients present in the daily diet, it is fat that provides the most significant amount of energy) among the respondents was checked (Table 7), assessing the responses on a 6-point scale, and assigning higher points to a higher frequency, i.e., for responses “never/almost never” 1 point was assigned, and for the answer “several times a day” 6 points were assigned. The frequency of consumption of good-quality fats and representatives of fat products, which, according to the recommendations, should be limited or eliminated, was examined.

The mixed group used meals prepared with deep fat more often and used butter or cream to add to the dishes. Vegans, as one supposed, used vegetable oils more frequently than people on a mixed diet. The differences in consumption between the groups are statistically significant (except for “meals with a large amount of fat added”).

The summary of the health conditions of the respondents (Table 8) shows a similar number of people with cardiovascular diseases and hypertension in their history. Risk factors for cardiovascular diseases taken together with lipid and carbohydrate disturbances showed that the OMN group had a higher risk of approximately 8 p.p. The VEGE group showed a 5 p.p. higher risk associated with tobacco smoking and alcohol drinking. Considering that the number of obese people in the OMN group was twice as high as in the VEGE group, their health conditions show worse results than in the VEGE group.

### 3.3. Changes in Diets and Quality of Life Caused by the Epidemic Situation

#### 3.3.1. Quality Life Change

In both groups, the overwhelming number of respondents (74% OMN vs. 73.7% VEGE) replied that the epidemic situation changed their daily quality of life. Most respondents declared that the damaging information about the number of cases and deaths from COVID-19 hurt their well-being (OMN 60.3% vs. VEGE 62.4%). The most common emotions evoked by the epidemic situation were the three declared descriptions of well-being:

- “I feel bad because of the need to isolate and maintain social distancing.”
- “Powerlessness, helplessness.”
- “Anger.”

Approximately 60% of respondents changed their physical activity during the pandemic. Overall, more people decreased (OMN 37% vs. VEGE 33.9%) their physical activity than increased (OMN 19.4% vs. 23.2%) during the pandemic. The physical activity during the prevailing epidemic situation changed among the respondents as follows:

- The highest number of responses was obtained for “the level of physical activity was at an average level, and during the pandemic, physical activity decreased” (22.7% OMN vs. 20.2% VEGE).
- The answer “the level of physical activity was at an average level and remained at this level” was indicated by 18.4% of OMN vs. 18.8% VEGE.
- The answer “the level of physical activity was at a low level and remained so” was chosen by 20% of people on a mixed diet and 18.5% of vegetarians.

Changes in the sleep quality of the respondents were checked using a multiple-choice question, where the respondents could choose adequate declarations; the most frequently chosen answers were:

- “I sleep longer”—29% OMN vs. 30.1% VEGE.
- “I wake up later”—22% OMN vs. 18% VEGE.
- “I go to sleep later”—21.1% OMN vs. 16.7% VEGE.
- “I go to bed at less regular times”—18% OMN vs. 16.1% VEGE.

The changes in the sleep duration of the respondents were examined by asking what this aspect looked like before and during the pandemic. It was shown that in the mixed group, 6.7 p.p. fewer people declared sleep <5–6 h, and 18.4 p.p. more people declared rest >8 h during the pandemic compared to the situation before it. During the pandemic, the situation for the vegans was very similar, and the differences were equal to 9.9 p.p. and 17 p.p., respectively.

Over a quarter of the respondents indicated that they were working more due to the situation (29.2% OMN vs. 26.3% VEGE).

#### 3.3.2. Changes Related to the Number of Meals Eaten, Dietary Preferences, and Body Weight Value

**Figure 1:**
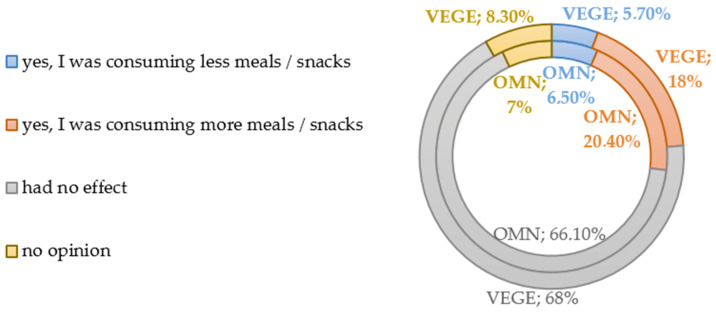
Changes in consumption of food during the COVID-19 pandemic.

Most of the respondents in both groups declared that the incoming information related to the pandemic (on the number of cases and deaths) did not affect food consumption (OMN 66.1% vs. 68% VEGE). A quarter of responders reported a change in meals and snack consumption; a certain percentage of the OMN (20.4%) or VEGE (18%) group reported an increased consumption; the results are presented in Figure 1.

Concerning changes in dietary preferences, where respondents could choose multiple declarations, vegetarians most often indicated eating more healthy meals/products, including fruit and vegetables (30.6%), and increasing the consumption of sweet (25%) and salty (21.2%) snacks. People on a mixed diet indicated increased consumption of sweet snacks (29.9%), followed by healthy meals/products (26.2%), and to a lesser extent, salty snacks (19.4%). In both groups, about 60% of respondents favored the changes in dietary preferences in the epidemic situation. The results are presented in Table 9.

**Figure 2:**
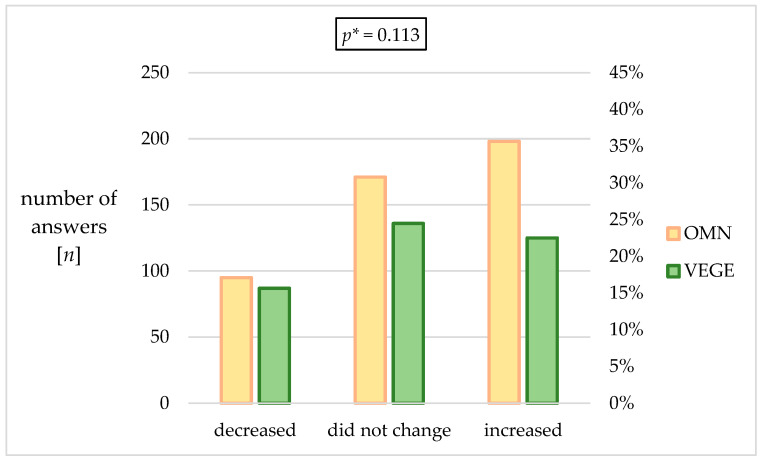
Changes in weight of the responders during the pandemic. * *p*—calculated between the studied groups using χ^2^ Tests.

It was checked whether the respondents declared a change in body weight due to the current epidemic situation. Approximately 5% of the respondents reported “did not check their body weight”; thus, they were excluded from the following calculations. The results are presented in Figure 2. The most significant percentage of respondents in the mixed group indicated weight gain during the pandemic (42.7%), which was 6.8 p.p. more frequently indicated than in the VEGE group. The statistical analysis results show no significant differences between the studied groups regarding changes in body weight.

### 3.4. Dietary Changes during a Pandemic—Refer to 60% of Responders Declaring Such Changes

Changes in the diet during the pandemic were confirmed by 59.7% of vegetarians and 60.7% of people on a mixed diet. The data described (in this section) concerns only those respondents who confirmed the occurrence of changes in eating due to the epidemic situation.

**Figure 3:**
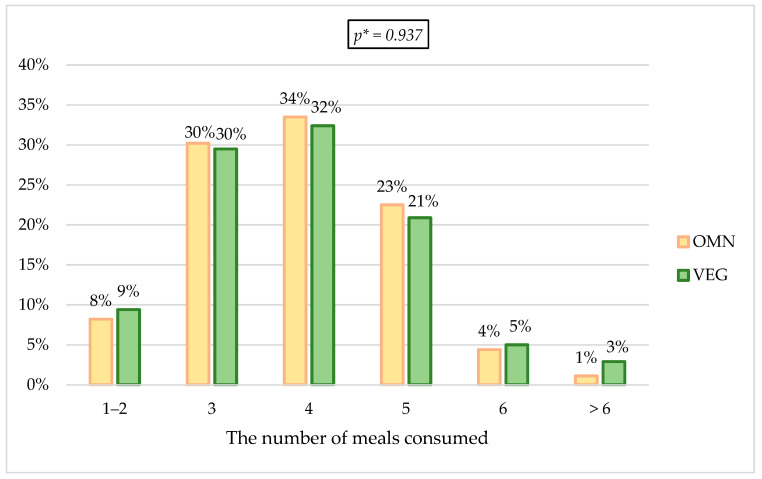
Changes in the number of meals before the pandemic. * *p*—calculated between the studied groups using Mann-Whitney U Test.

**Figure 4:**
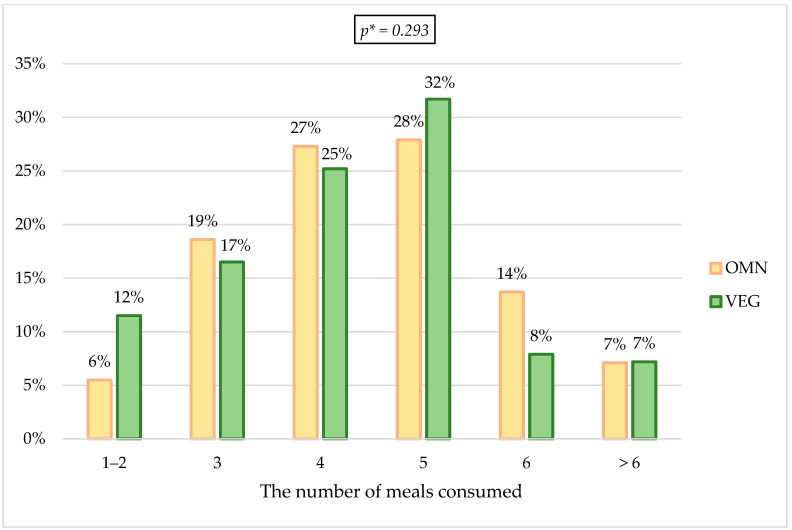
Changes in the number of meals during the pandemic. * *p*—calculated between the studied groups using Mann-Whitney U Test.

It was checked in detail how the frequency and the regularity of food intake changed in the epidemic situation. The changes in the amounts of consumed food among the respondents were checked; approximately 45% of the respondents in both groups declared an increase in the amount of food consumed daily, and the lowest percentage of responders consumed less food (18.89% VEGE vs. 15.2% OMN). As for changes in the number of meals during the day, in both groups, the highest percentage of indications indicated four meals before the pandemic (33.5% OMN vs. 32.4% VEGE). During the pandemic, in the VEGE group, 10.8 p.p. more respondents declared eating five meals a day; in the OMN group, this change was 5.4 p.p. according to the time before the pandemic. The percentage of responses indicating the consumption of three meals a day decreased by almost half (Figure 3 and Figure 4).

Regarding the regularity of consumed meals, 45.7% of the VEGE group indicated that they consumed more regular meals during the pandemic; the percentage in the mixed group was lower and amounted to 36.8%. The most common response in terms of eating regularity in the OMN group was the change in food consumption regularity during the pandemic—41.6% OMN vs. 32.6% VEGE.

More frequent snacking between main meals was more often observed in the OMN group (56.3%) than in the VEGE group (49.8%); the obtained result shows that approximately half of the respondents in both groups declared an increase in the frequency of snacking during the pandemic.

Changes in the way of preparing meals show that 40% of the respondents in both groups declare to pay more attention to self-preparation of meals, approximately 22% of the respondents consume more ready meals/products.

From the mixed group, 69.2% of people, and 73.1% of vegetarians had an increased frequency of using ready-made food products during a pandemic.

The respondents were asked whether the consumption of products from the listed food categories increased during the pandemic and to estimate the portions consumed during the day (example portions were included in the question). The answers were assigned numerical values: “No [product] consumption has changed” = 0; “I don’t like [product]” = 0; “Yes, by about 1–2 units/day (compared to the period before the pandemic)” = 1; “Yes, I use them very often (compared to the period before the pandemic, I increased my consumption by> 3 items/day)” = 2, based on which statistical analysis was performed, in terms of the significance of differences in the obtained results.

The results showed significant differences in the consumption of vegetables, legumes, and dairy products or their plant substitutes; they were consumed significantly more often in the VEGE group compared to the OMN group; the results are presented in Table 10.

Additionally, 26.4% of vegetarians and 22.5% of the OMN group declared an increase in alcohol consumption. In turn, 28.2% of vegetarians reported a complete abandonment of alcohol consumption, compared to 22.5% of people on a mixed diet.

An increased number of cigarettes smoked or starting smoking during the pandemic was declared by 14% of people on a mixed diet and 15.6% of vegetarians; approximately 80% of respondents stated that they had not smoked before the pandemic and did not smoke at the time of the survey.

**Figure 5:**
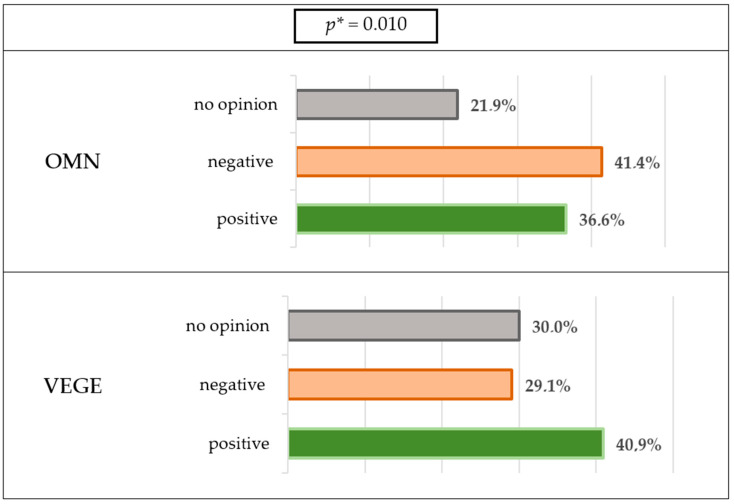
Dietary changes during the lockdown. * *p*—calculated between the studied groups using χ^2^ Tests.

For 41.4% of people on a mixed diet, they declared that changes in their eating were negative. In comparison, in the case of vegetarians, for 40.9%, they were positive (Figure 5), which shows statistically significant differences.

Factors influencing the diet of the respondents were checked by selecting declarations; the most frequently chosen answers were:

- More time for snacks/snacking—48.9% OMN vs. 38.8% VEGE.
- More time to prepare meals—35.8% OMN vs. 34.5% VEGE.
- I wanted to take better care of my health in this particular period—31.6% OMN vs. 33.5% VEGE.
- Stress, fear, negative emotions—29.8% OMN vs. 28.2% VEGE.

## 4. Discussion

The study showed distinct differences in behavior changes that are components of quality of life, especially in nutrition aspects, between the studied groups from the same environment, adhering to different dietary patterns during a particular situation, such as the pandemic.

### 4.1. Risk Factors of Cardiovascular Diseases among the Respondents (Characteristics of the Study Group)

#### 4.1.1. Classification by BMI Value

The division according to the BMI showed that overweight and obesity were more common in the OMN group (obesity more than twice as often), and the BMI value was significantly higher compared to the VEGE group. However, it should be emphasized that the percentage of these people was <8% for obesity and <20% for overweight. These observations are consistent with the results of numerous studies [49,52,54,60,79,80,81,82,83,84,85].

#### 4.1.2. Disease Burden—Cardiovascular Risk

A similar burden of the disease characterized the study groups. The results showed almost 50% CVD burden in both groups, which may seem surprising. A higher burden of these diseases is observed in Central and Eastern European (CEE) countries compared with Northern, Southern, and Western European states [86,87]. According to the Central Statistical Office in Poland, in 2020 cardiovascular diseases are the leading cause of deaths, accounting for 40% of deaths [88]. The data shows that for every 100,000 of the country’s population, 455 died due to CVD [88]. It should be noted that a family CVD history is an essential factor; however, in most cases, the family burden is a duplication of the parents’ unhealthy lifestyle, as hypertension, diabetes, lipid abnormalities, and excessive weight appear due to an unhealthy diet and low physical activity [86,89,90,91].

Most of the respondents showed normal blood pressure levels, cigarette smoking was significantly more common in the VEGE group, but most did not smoke in general. Both groups reported the highest percentage of low consumption of alcohol (approximately from 65–68%), while about 20% of responders reported complete alcohol withdrawal. Concerning the study by Lin et al., results differ from claims for alcohol consumption and smoking, while comparisons to health burdens show agreement with the results of this work in some respects [92]. The respondents in the cited study indicated the answer “never” as the most frequent answer regarding alcohol consumption (about 85%) and did not show any differences in the responses regarding smoking. The results of a study by Lin et al., showed significantly more frequent smoking among the VEGE group; vegetarians (*n* = 2166) had substantially lower medical expenditure on hypertension (28% lower) and dyslipidemia (31% lower) compared to omnivores (*n* = 4332). The results with regard to coronary heart diseases (22% lower) were not statistically significant [92].

In this study, hypertension among the respondents was at the same level. It did not show statistically significant differences between the study groups or consider the division by sex. At the same time, the negative response to the presence of abnormal values of the lipid profile was reported more often by people from the VEGE group (76.08%) compared to the OMN (73.82%). Similar observations are also confirmed by the results of the study by Jakše et al., comparing the risk of cardiovascular diseases in vegans (*n* = 51) and non-vegans (*n* = 29) [93]. The groups based on lipid profile and blood pressure values showed significant differences in the results. They indicated potentially better cardiovascular health in the vegan group, resulting in significantly lower lipid levels (except HDL cholesterol) and blood pressure than in non-vegans. Another study (online survey on mental health and multidimensional lifestyle behaviors during home confinement—ECLB-COVID-19) conducted during the pandemic, with respondents from different countries (*n* = 1047), with a division according to the “health state”, showed that the number of people with risk factors for cardiovascular disease was 7.7% [28]. Comparing the results with our study, the percentage of people at risk is higher, taking into account the following factors: smoking regularly and/or medium/high alcohol consumption—the VEGE group shows a higher risk (VEGE 24.46% vs. OMN 19.4%). On the other hand, dyslipidemias and/or dysfunction of carbohydrate metabolism indicate a higher risk for people from the OMN group (OMN 25.5% vs. VEGE 17.74%). Possible differences between the cited work and these studies may result from the considered various risk factors, different places of residence, age differences, or a smaller size of the surveyed groups.

Considering the percentage of people with cardiovascular risk factors and the fact that the number of obese people in the OMN group was twice as high as in the VEGE group, their health conditions show worse results than in the VEGE group, which shows some similarity to the results obtained in the work of Lin et al., where the omnivores group required higher health expenditure than the vegetarian group [92]. According to numerous reports, the consumption of red meat increases the risk of mortality, cardiovascular diseases, cancer, and type 2 diabetes, and vegetarian diets have a beneficial effect on shaping health, as mentioned in the introduction [94,95,96,97,98,99,100,101,102].

#### 4.1.3. Nutritional Factors Important in Estimating Cardiovascular Risk

A poor-quality diet with a high supply of fats, high in saturated fatty acids (SFA), and low in polyunsaturated fatty acids (PUFA) is not recommended and is described as one of the leading causes of cardiovascular diseases [41,103,104,105,106,107]. People from the VEGE group showed more ideal nutritional behavior concerning the checked frequency of consumption of products from this food category, showing a significantly higher frequency of consumption of vegetable oils. The OMN group, on the other hand, significantly more often declared eating “meals prepared in deep fat” and adding “butter or cream” to the meals. According to global recommendations, one should limit the consumption of animal fats and choose plant-based sources instead [41,107,108]. The frequency of fat consumption in the OMN group shows discrepancies with the recommendations, and the results of the VEGE group prove their advantage.

### 4.2. Changes in the Quality of Life Caused by an Epidemic Situation

Numerous observations were obtained regarding the changes in the quality of life among the respondents, common for both the OMN and VEGE groups. Most of the respondents, approximately 74%, declared a change in their daily quality of life, and approximately 60%, a negative impact on their well-being, where the most common emotions were discomfort due to the necessary isolation, helplessness, and anger. Nochaiwong et al., in a systematic review and meta-analysis, revealed the global prevalence of depression at a level of 28%, 26.9% for anxiety, 36.5% for stress, and 50.0% for psychological distress [109]. The last is similar to the value obtained in this study.

#### 4.2.1. Changes in the Quality of Life—Physical Activity

In total, approximately 60% of respondents changed their physical activity during the pandemic, which was confirmed by the results of Matsungo et al. [30], where 62.5% of the respondents (*n* = 507) declared a decrease in their physical activity, and the results of Rodriguez-Perez et al. [25] showed this was the case for over 59.6% of the respondents (*n* = 7514). In this study, the VEGE group reported an increase in physical activity by 3.8 p.p. more often than the OMN group. Moreover, the VEGE group declared a reduction in physical activity by 3.1 p.p. less frequently than the OMN group. Results similar to those obtained in the OMN group (OMN 37% vs. VEGE 33.9%), in terms of reduction in physical activity, were shown in 38% of the subjects (*n* = 1047) in the study by Ammar et al. [28]. Observations of a decrease in physical activity among approximately between 40 and 50% of respondents confirm the results of other studies from various countries [5,34,110,111,112]. Referring to the cited sources, one could presume that the reduction in physical activity in the group of vegetarians is lower than the results presented in the available literature, where people who are traditionally well-nourished are taken into account. Yücel et al., as the only similar study, also assess pandemic changes compared among vegetarians (*n* = 357) (including vegans, *n* = 246) and omnivores (*n* = 362) [113]. In the cited study, very similar results regarding physical activity among the studied groups were shown (which is in line with the results obtained in this study); additionally, the physical activity durations of vegetarian, vegan, and omnivorous individuals decreased significantly in comparison to the pre-pandemic level by approximately 60%. This result differs from that obtained in this study (the estimated decrease in activity is approximately between 20 and 30%), possibly because of the higher health awareness of the respondents and because the research was conducted at a later stage of the pandemic (approximately a one year difference in the recruitment of participants), so it can be presumed that as the pandemic progressed, people returned to their good old habits or wanted to take better care of their health by increasing physical activity.

#### 4.2.2. Changes in Quality of Life—Sleeping

The most frequently chosen statements regarding the quality of sleeping of the respondents were consistent in the studied groups. They indicated that approximately 30% of the respondents slept longer, approximately 20% got up later, approximately 17% of the respondents declared less regular sleep times, and no differences in sleep quality were observed between the study groups. The observations from this work confirm the results of the study by Marelli et al., where significant differences due to the pandemic were observed; subjects (*n* = 400) went to sleep later, woke up later, and also showed sleep latency [114]. The results of other studies confirm the occurrence of changes in the quality of sleep [115,116,117]. The systematic review and meta-analysis by Nochaiwong et al., showed the global prevalence of sleep problems at the level of 27.6% (*n* = 398,771) [109]. Piekarska et al., observed changes in over 1/3 of respondents (36.04%, *n* = 1956) [115]. Additionally, in this study, non-vegetarians (*n* = 1209) suffered from insomnia or sleepiness more often than vegetarians (*n* = 747). The results of the survey by Yücel et al., show significant differences in the observations of sleep duration [113]. The group of omnivores increased sleep time in the greatest proportion (54.4%) compared to vegetarians (40.9%) or vegans (41.9%) [113].

#### 4.2.3. Changes in Quality of Life—General Eating Behavior

Responses concerning the studied changes in general eating behavior showed that in almost 67% of respondents, information related to the pandemic did not affect the number of meals consumed; on the other hand, an increase in meals and snacks was reported by approximately 20% (18% VEGE vs. 20.4% OMN). A systematic review (2021) by Khan et al., shows that most studies show an increase in food intake associated with increased snacking [76].

Subsequent responses to the habits of the respondents show some differences between the groups. The VEGE group indicated the highest percentage of responses indicating an increase in the consumption of healthy meals and products, including fruit and vegetables and whole grain cereal products (VEGE 30.6% vs. OMN 26.2%), while the OMN group most often indicated that the consumption of sweet snacks increased (OMN 29.9% vs. VEGE 25%). Approximately 20% of the respondents in both groups declared an increase in the consumption of salty snacks, as well as approximately 18% unhealthy products (ready-made products and instant meals). In a cohort study by Ruiz-Roso et al., a significant increase in the consumption of sweet food by 20.7% and fried food during COVID-19 confinement was shown among the respondents (*n* = 820) [118]. Less than 1/3 of respondents from a systematic review by Khan et al., (*n* = 469,362) showed both positive “consumption of fresh fruits and vegetables on a daily basis” and the same number (less than 1/3) negative behaviors with the “consumption of sweets and desserts every day” during confinement [76], which is similar to the results from this study, although 5% more people from the VEGE group indicated correct eating behavior. Similar observations were shown in the survey by Yücel et al., where the organic food consumption of vegetarian and vegan individuals increased significantly in comparison to the omnivore group [113]. Their consumption of fast food and frozen food decreased, and in the case of vegans also, “junk food” consumption decreased compared to omnivores.

#### 4.2.4. Changes in Quality of Life—Effects on Body Weight

The OMN group showed a higher percentage of people declaring weight gain (OMN 42.7% vs. VEGE 35.9%), while the VEGE group most often indicated the answer “my body weight did not change” (VEGE 39.1% vs. OMN 36.9%). In the VEGE group, on the other hand, weight loss was declared approximately 5 p.p. more often than in the OMN group. In the mentioned systematic review by Khan et al., all studies that showed increased food intake, including snacks, also showed a consequence of increased body weight [76].

A study by Yücel et al., accounting for vegetarians, vegans, and omnivores, shows a high agreement with our research regarding a higher percentage of weight gain during the pandemic among omnivores (42.3%) than among vegetarians (35.9%) and vegans (39.8%) [113]. The observation also confirmed that in the group of vegetarians, the most common response was the declaration of no change in body weight (39.2%), while in the omnivores group, this result was at the level of 32%. Additionally, also about 6 p.p. less frequently in the omnivores group was a decrease in body weight. The group with the highest increase in body weight and sleep duration was the omnivores (*p* < 0.01) [113].

### 4.3. Additional Detailed Observations Regarding Changes in Eating Habits during the Pandemic—Concerns about 60% of Respondents Who Confirmed the Existence of Differences

The occurrence of changes in eating habits was confirmed by approximately 60% of the respondents.

#### 4.3.1. Changes in Food Intake

Approximately 45% of the respondents declared an increase in the amount of food consumed during the day. In addition, the results of numerous studies presented in the systematic review by Khan et al., confirmed these observations [76]. In another review examining the impact of the pandemic on changes in eating habits from the same year (2021) by Bennett et al. [3], 9 out of 23 analyzed studies (3 studies are also cited by Khan et al.) from different countries also confirmed a significant increase in snacking frequency. Usually, snacking appeared in the evening/at night and was regarded as reaching for sweets or the so-called “comfort food” and/or was a consequence of eating after stressful situations [3,76]. The results of the study by Yücel et al., where changes in habits between vegetarians, vegans, and omnivores were studied, showed that snack consumption of vegetarian individuals significantly increased when compared to omnivores (*p* < 0.05). It is not in line with the results of this study, as no differences were observed between the respondents in this regard [113].

#### 4.3.2. Changes in the Number of Meals Eaten

Among people confirming changes, differences can be observed between the studied groups in terms of the number of meals during the day. Before the pandemic, both groups indicated the consumption of four meals a day most often (approximately 33%), while during the pandemic, the most frequently chosen answer in both groups was eating five meals a day (31.7% of persons from the VEGE group and 27.9% participants from the OMN group). Most studies show an increase in the number of meals consumed [7,21,24,31,110,119,120,121,122,123,124], and some showed no difference in the number of meals eaten during the lockdown period [23,25,109]. Study results by Yücel et al., are consistent with the observations of this work—an increased number of meals during the pandemic was reported in the group of vegetarians (but not in the group of omnivores) [113]. Eating four or five meals a day is beneficial and in line with the recommendations [107,125]; however, only if it does not go hand in hand with increasing the consumption of unhealthy snacks, causing the individual’s energy requirement to be exceeded. Increasing the consumption of meals may show beneficial consequences, e.g., increasing the consumption of fruit and vegetables (e.g., in the form of a salad) or nuts as a snack. Regular food intake is also vital for health.

#### 4.3.3. Changes in the Regularity of Meals Eaten

The VEGE group ate more regularly during the pandemic, as this was the answer indicated by most respondents (VEGE 45.7% vs. OMN 36.8%). This response significantly differed from the response of the OMN group, which, in the most significant percentage, chose a response indicating less regular food consumption (OMN 41.6% vs. VEGE 32.6%) during the pandemic. The VEGE group also showed a lower frequency (by 6.5 p.p.) of more snacking during the pandemic than the OMN group; however, it was revealed that approximately 50% of respondents in both groups declared an increased frequency of snacking. The study by Górska et al., where the regularity of consumption was checked in five different countries, shows mainly an increase in the regularity of meals consumed during the pandemic [126]. The results of the cited work show the differences related to a place of residence and age. To the best of our knowledge, there is no other research comparing the regularity of consumed meals in vegetarian groups during the pandemic.

#### 4.3.4. Changes to the Way Food Is Prepared

Both positive and negative consequences of the epidemic situation were observed in the eating behavior regarding the way of preparing meals in both groups, showing no differences between the studied groups. Of the respondents, 40% declared to pay more attention to preparing meals on their own. The results of research from a systematic review by Khan et al., show an increase from 40% to 62% in the number of people involved in cooking and an increase in the consumption of homemade recipes [76].

In addition, among the respondents in this study, negative consequences were observed in the form of eating more ready meals/products as the main meals consumed and more frequent use of ready-made food products by approximately 70% of respondents. Numerous studies from a review by Khan et al., showed increased consumption of high-density processed foods [76].

#### 4.3.5. Changes in Nutritional Behavior (Favorable or Unfavorable)

Among the respondents, it was checked how the frequency of consumption of selected food categories changed. Better results concerning the changes in habits, confirmed by more frequent consumption of the studied groups of products, were observed in the VEGE group. The results showed significantly more frequently selected products, i.e., vegetables (*p* = 0.029), legumes (*p* < 0.001), and dairy products or their plant substitutes (*p* = 0.002), in the VEGE group compared to the OMN group. In terms of increasing the frequency of consumption, other food categories, such as fruit, wholegrain cereal products, and nuts and seeds, obtained a similar percentage distribution of responses in both groups. There was an approximately between 30% and 35% increase in their consumption during the pandemic. In a study by Durán-Agüero et al., the results also showed an increase in legume consumption (54.5%) in the vegetarians/vegans compared to non-vegetarians (a rise of 25%) [127], showing very similar observations to this study—VEGE increased consumption by 44.1%, while the OMN group did so by 23.5%. The increased consumption of pods in these groups may be because legumes are often the staple of vegetarian/vegan main dishes. Regarding the consumption of fruit and vegetables, observations from the systematic review by Khan et al., indicate that less than 1/3 of the respondents consume fruit and vegetables daily, emphasizing that a similar percentage of respondents declare the daily consumption of sweets and desserts [76].

#### 4.3.6. Pandemic Impact—Summary of Changes in the Nutrition Quality

Of the people on a mixed diet, 41.4% declared that changes in eating behaviors were negative, while for vegetarians 40.9% were positive, which is confirmed by the observations described above. Taking into account the previously cited studies (reviews by Khan et al. and Bennett et al.), both favorable and unfavorable consequences have been shown, and their impact may be short- or long-term on health [3,76]. The studies cited in the systematic review included people eating traditionally (not giving up consumption of animal and/or animal-origins products) [3,76]. Our study comparison in two different eating groups revealed that the VEGE group showed more positive eating behaviors and also more positive consequences of dietary changes during the pandemic. The only study that compares the pandemic’s impact on vegetarian and traditional groups, by Yücel et al., also showed more regularities in the vegetarian groups [113].

It is well-known that people who switch to vegetarianism pay more attention to the products and meals they eat, choosing natural products while adhering to the eco-friendly principle, which has a beneficial effect on health [60,128]. Our research confirms such observations, but it is necessary to emphasize that this applies to people who care about a proper balance of such a diet and choose healthy products of good quality. The available literature lacks comparisons of the impact of the pandemic/lockdown on non-traditional feeding groups. A study showed that people on such diets might have a lower risk of developing severe COVID-19 symptoms; hence, such diets may take place in the recommendations, both as a preventive action against civilization/cardiovascular diseases, but also concerning COVID-19 [129]. It seems necessary to conduct more extensive research, including numerous groups of vegetarians, including vegans, to discuss this possibility more deeply.

### 4.4. Strengths and Limitations

#### 4.4.1. Strengths

To the best of our knowledge, this is the first study to consider changes during the pandemic among people on a vegetarian (including vegan) diet and traditional diets regarding the quality of life changes, including a thorough examination of eating habits.

- The study participants did not show any differences concerning average age, which proves reliable samples. The division of the study participants into age categories was primarily planned, but due to the above, such a division was abandoned. The similar age in groups may be due to the place of recruitment, social media, which young adults or adults mainly use.
- The results obtained in this study enrich the current positive reports on the vegetarian diet showing that even under a situation that has never happened before (the pandemic), people who follow such a diet show more correct eating behavior, which can have a positive effect on health.

#### 4.4.2. Limitations

- The measurement was not previously validated in the literature.
- A narrow age group does not give a complete cross-section of possible outcomes.
- The data were collected through an online-based questionnaire; the possibility of selection bias should be taken into account, as it is possible that people who completed our form were interested in a healthy lifestyle.
- Since a survey was performed during the lockdown, the possibilities of checking the nutritional status were limited. Moreover, most of the available studies comparing groups of vegetarians and non-vegetarians also took into account the BMI indicator in question, so it was concluded that its use might constitute a valuable reference. However, it should be noted that the limitations of BMI are being increasingly emphasized; therefore, it should not be considered a perfect tool [49,130].
- Respondents may have been tempted to report increased consumption of commonly considered “healthy” foods and consumption of “unhealthy” food as lower than it is.
- The fact that the questionnaire was web-based and self-reported could have negatively influenced data quality.
- This study was based on univariate analysis.

## 5. Conclusions

### 5.1. Information on Cardiovascular Risk Factors

The study participants showed similar results in terms of the disease burden. Most of the respondents did not indicate any abnormalities characteristic of cardiovascular diseases or hypertension; incorrect carbohydrate metabolism values were more common in people on a mixed diet (the difference equals 6.4 p.p., *p* = 0.010). Risk factors for CVD taken together with lipid and carbohydrate disturbances showed that the OMN group, compared to the VEGE, had a higher risk of approximately 8 p.p., while the VEGE group, compared to the OMN, showed a 5 p.p. higher risk associated with tobacco smoking and alcohol drinking. Considering that the number of obese people in the OMN group was twice as high as in the VEGE group, their health conditions show worse results than in the VEGE group. People from the VEGE group showed correct nutritional behavior concerning the frequency of consumption of the tested products.

### 5.2. Information on Changes in Quality of Life

The obtained findings about changes in quality of life caused by epidemic situations impacted approximately 74% of responders (mainly a negative impact on their well-being—OMN 60.3% vs. VEGE 62.4%). There was no difference between the respondents’ responses in terms of sleep quality, which showed that subjects slept longer, woke up later, or showed less regular sleep patterns. Approximately 60% of respondents changed their physical activity during the pandemic.

An increase in the intake of meals and snacks was declared by approximately 20% of the respondents. The VEGE group showed more correct eating behavior than the OMN group.

The OMN group showed a higher percentage of people declaring weight gain (OMN 42.7% vs. VEGE 35.9%).

### 5.3. Changes in Eating Habits during the Pandemic

The VEGE group indicated that they were more regular with their meals, snacked less often, and were more likely to eat healthier foods than the OMN group. A comparison of two different dietary groups living in the same area showed that the VEGE group exhibited more positive eating behaviors and more often declared positive consequences of dietary changes (40.9%) during the pandemic than the OMN group (41.4%) declared dietary changes were negative.

## Figures and Tables

**Table 1:** Characteristics of the study groups.

Characteristics *n* = 861	OMN	VEGE
	*n* (%) *	
The gender division, *p* *^1^ = 0.009		
Participants	489 (56.8)	372 (43.2)
Female	420 (85.9)	341 (91.7)
Male	69 (14.1)	31 (8.3)
Educational level, *p* *^1^ = 0.025		
University or university college level	440 (90.9)	315 (84.9)
Secondary education	36 (7.4)	39 (10.5)
Vocational education	5 (1.0)	10 (2.7)
Primary education	3 (0.6)	7 (1.9)
Place of residence, *p* *^1^ = 0.001		
Inhabiting a city The following responses were considered: a city with up to 50,000/100,000/250,000 residents; a city with more than 250,000 residents.	384 (78.5)	324 (87.1)
Rural area	105 (21.5)	48 (12.9)
Age, years, *p* *^2^ = 0.744	**mean ± SD**	
all groups	28.6 ± 7.60	29.2 ± 8.68
Female	29.0 ± 7.49	29.1 ± 8.77
Male	26.6 ± 7.99	30.6 ± 7.72

* *n* (%)—the number of participants and their percentage content in the study group. *^1^ calculated between the studied groups using χ^2^ Tests. *^2^ calculated between the studied groups using Mann–Whitney U tests.

**Table 2:** Family history of cardiometabolic disease.

	Possible Answer	Cardiovascular Diseases, i.e., Heart Disease, Heart Attack, Stroke, Arterial Hypertension, Thrombosis		Abnormal Values of the Lipid Profile (Total Cholesterol, Triglycerides, HDL Cholesterol, LDL Cholesterol)		Fasting Glucose Abnormalities/ Diabetes/Insulin Resistance	
Groups							
		*n* *	% *	*n* *	% *	*n* *	% *
**Yes**							
OMN		243	49.69	256	52.35	167	34.15
VEGE		175	47.04	175	47.04	133	35.75
**No**							
OMN		225	46.01	157	32.11	293	59.92
VEGE		178	47.85	116	31.18	210	56.45
**I don’t know**							
OMN		21	4.29	76	15.54	29	5.93
VEGE		19	5.11	81	21.77	29	7.80
*p* *^2^		0.684		0.057		0.431	

* *n*, %—the number of participants and their percentage content in the study group. *^2^ *p*—calculated between the studied groups using χ^2^ Tests.

**Table 3:** Medical conditions of the respondents.

	Possible Answer	Cardiovascular Diseases, i.e., Heart Disease, Heart Attack, Stroke, Arterial Hypertension, Thrombosis		Abnormal Values of the Lipid Profile (Total Cholesterol, Triglycerides, HDL Cholesterol, LDL Cholesterol)		Fasting Glucose Abnormalities/ Diabetes/Insulin Resistance	
Groups							
		*n* *	% *	*n* *	% *	*n* *	% *
**Yes**							
OMN		31	6.3	57	11.7	64	13.1
VEGE		24	6.5	40	10.8	25	6.7
**No**							
OMN		444	90.8	361	73.8	390	79.8
VEGE		334	89.8	283	76.1	318	85.8
**I don’t know**							
OMN		14	2.9	71	14.5	35	7.2
VEGE		14	3.8	49	13.2	28	7.5
*p* *^2^		0.758		0.751		0.010	

* *n*, %—the number of participants and their percentage content in the study group. *^2^ *p*—calculated between the studied groups using χ^2^ Tests.

**Table 4:** Characteristics of the cardiovascular disease risk factors.

	CD Risk Factors:	The Blood Pressure Characteristic *p* = 0.864 *^1^		Tobacco Smoking *p* = 0.003 *^2^		Alcohol Consumption *p* = 0.448 *^3^	
Studied Groups:		*n* *	% *	*n* *	% *	*n* *	% *
		**I don’t know**		**Yes, occasionally**		**High consumption** **(daily or 3–5 x week)**	
OMN		52	10.6	40	8.18	19	3.9
VEGE		52	14.0	46	12.37	25	6.7
		**I do not measure**		**Yes, regularly**		**Medium consumption** **(1–2 x week)**	
OMN		70	14.3	28	5.73	48	9.8
VEGE		60	16.1	34	9.14	32	8.6
		**<120/60 mmHg**		**No**		**Low consumption (once a month or less, several times a month)**	
OMN		146	29.9	421	86.09	334	68.3
VEGE		127	34.1	292	78.49	241	64.8
		**140/80–120/60 mmHg**				**None**	
OMN		201	41.1			86	17.6
VEGE		118	31.7			73	19.6
		**>140/80 mmHg**				**No answer**	
OMN		20	4.1			2	0.4
VEGE		15	4.0			1	0.3

* *n*, %—the number of participants and their percentage content in the study group. *^1^ *p*—calculated between the studied groups using χ^2^ Tests. The number assigned for given answers about blood pressure used for calculating *p* values includes 0—for incorrect values, 1—for correct values (for female *p* = 0.858, for male *p* = 0.790). The answers “I don’t know” and “do not measure” were not included in the significance test. *^2^ *p*—calculated between the studied groups using Mann–Whitney U Tests. The number assigned for given answers about smoking used for calculating *p* values includes 0—“no smoking”, 1—“smoking occasionally”, 2—“smoking regularly”. *^3^ *p*—calculated between the studied groups using Mann–Whitney U Tests. The number assigned for given answers about alcohol consumption used for calculating *p* values includes 0—“no alcohol consumption”, 1—“once in a month or less”, 2—“several times a month”, 3—“approx. 1–2 times a week”, 4—“approx. 3–5 times a week”, 5—“everyday”.

**Table 5:** The mean BMI value of the respondents.

Categorization	OMN BMI [kg/m^2^] Mean ± SD		VEGE BMI [kg/m^2^] Mean ± SD	
all groups p * < 0.001	23.5 ± 4.89		21.8 ± 3.57	
by gender	Female	Male	Female	Male
	23.3 ± 5.01	24.7 ± 3.94	21.6 ± 3.53	23.6 ± 3.54

* *p*—calculated between the studied groups using Mann–Whitney U Tests.

**Table 6:** The classification of the studied groups concerning the BMI index.

BMI Classification *p* < 0.001 *^2^	Group	*n* *	% *
Underweight (<18.5 kg/m^2^)	OMN	32	6.6
	VEGE	49	13.2
Normal weight (18.5 to <25.0 kg/m^2^)	OMN	325	66.9
	VEGE	263	70.7
Overweight (≥25.0 to <30.0 kg/m^2^)	OMN	95	19.5
	VEGE	51	13.7
Obesity (≥30 kg/m^2^)	OMN	34	7.0
	VEGE	9	2.4

* *n*, %—the number of participants and their percentage content in the study group. ^*2^ *p*—calculated between the studied groups using χ^2^ Tests.

**Table 7:** The frequency of consumption of products/meals that are a source of fat.

Responses	OMN	VEGE	p *
deep fried meals	2.28	2	<0.001
meals with the addition of a large amount of fat (fast-food, pizza, ready meals)	2.55	2.47	0.324
addition to meals, e.g., butter or cream (or vegetable substitutes for these products)	3.16	2.55	<0.001
oil addition to meals (rapeseed, sunflower, olive oil, linseed oil, etc.)	4.04	4.19	0.006

* *p*—calculated between the studied groups using Mann-Whitney U Test.

**Table 8:** Summary of the health state of the studied groups.

Health State of the Studied Groups	OMN		VEGE	
	*n*	%	*n*	%
With risk factors for cardiovascular diseases (smoking regularly and/or medium/high alcohol consumption)	95	19.4	91	24.46
With risk factors for cardiovascular diseases (dyslipidemias and/or dysfunction of carbohydrate metabolism)	125	25.5	66	17.74
With cardiovascular diseases	31	6.34	24	6.45
With hypertension (>140/80 mmHg)	20	4.1	15	4.0
With obesity (BMI ≥ 30 kg/m^2^)	36	7.4	9	2.4

**Table 9:** Changes in eating habits among the study groups—the most frequently indicated answers.

Responses p * = 0.360	OMN	VEGE
	%	
My dietary habits did not change.	41.8	40.3
Yes, I consumed more healthy products, such as fruits, vegetables, nuts, and whole grain products.	27.3	31.7
Yes, I consumed more sweets.	31.1	25.8
Yes, I consumed more savory snacks.	20.3	21.9
Yes, I ate more unhealthy meals/snacks, such as ready-made products, instant soups, dishes, and chips.	19.2	17.8

* *p*—approximated value for multiple-response data using χ^2^ Tests.

**Table 10:** Increasing product consumption during a pandemic, together with estimating the daily portion consumed.

Category of Products	% of Respondents Declaring Product Consumption Increase		Increase Consumption Measure [Mean ± SD]	p *^2^
	The Most Frequently Indicated Number of Servings			
	OMN	VEGE		
**Vegetables** (1 portion = a glass of vegetables or half a glass of vegetable juice)	36.5%	43.4%	OMN < VEGE * 0.481 ± 0.695 vs. 0.652 ± 0.815	0.029
	1–2 portion(s) (44.9%) 3 portions (26.5%) 4–6 portions (13.9%)	1–2 portion(s) (27.9%) 3 portions (29.7%) 4–6 portions (28.8%)		
**Fruits** (1 portion = 1 medium piece or half glass of fruit juice)	34.2%	35.9%	OMN < VEGE * 0.430 ± 0.651 vs. 0.491 ± 0.718	0.476
	<1 portion (16.7%) 1–2 portion(s) (51.5%) 3 portions (22.2%) 4–6 portions (6.5%)	<1 portion (15.2%) 1–2 portion(s) (49.3%) 3 portions (20.7%) 4–6 portions (9.7%)		
**Legumes** (1 portion = half a cup of legumes)	23.5%	44.1%	OMN < VEGE * 0.256 ± 0.482 vs. 0.545 ± 0.678	<0.001
	<1 portion (69.8%) 1–2 portion(s) (18.4%) 3 portions (3.5%)	<1 portion (36.7%) 1–2 portion(s) (40.4%) 3 portions (13.3%)		
**Whole grain products** (1 portion = 1 slice of bread or a half cup of groats	34.4%	34.7%	OMN > VEGE * 0.381 ± 0.559 vs. 0.379 ± 0.548	0.977
	<1 portion (19.6%) 1–2 portion(s) (37.8%) 3 portions (23%)	<1 portion (19.2%) 1–2 portion(s) (35.5%) 3 portions (23.8%)		
**Dairy products or plant-based substitutes** 1 portion = glass of milk	42.3%	57.5%	OMN < VEGE * 0.515 ± 0.660 vs. 0.676 ± 0.650	0.002
	<1 portion (24.2%) 1–2 portion(s) (44.7%) 3 portions (21.8%)	<1 portion (15.5%) 1–2 portion(s) (40.2%) 3 portions (27.4%)		
**Nuts, seeds** 1 portion = ¼ cup of nuts	32.7%	29.6%	OMN > VEGE * 0.372 ± 0.569 vs. 0.350 ± 0.581	0.512
	<1 portion (52.9%) 1–2 portion(s) (33.3%) 3 portions (7.2%)	<1 portion (52.8%) 1–2 portion(s) (33.2%) 3 portions (7.9%)		
**Meat** (only in the OMN group) 1 portion= 1 slice of ham or ½ chicken breast	22.1%	–	–	–

* In this column, the use of the symbols “>“, “<“ is meant to show more frequent consumption in one of the studied groups. *^2^ p—calculated by the Mann–Whitney U Test.

## Data Availability

All necessary data are included in the paper.

## Supplementary Materials

The following supporting information can be downloaded at: https://www.mdpi.com/article/10.3390/nu15020442/s1. Questionnaire S1: Nutrition habits of people on a vegetarian/vegan diet during the pandemic; Questionnaire S2: Nutrition habits of people on a traditional/mixed diet during the pandemic.
