# Supplementary material for: Dietary Habits and Lifestyle, Including Cardiovascular Risk among Vegetarians and Omnivores during the COVID-19 Pandemic in the Polish Population
Source: Nutrients. 2023 Jan 14;15(2):442. doi: 10.3390/nu15020442 (PMC9863812; doi:10.3390/nu15020442)
Supplement: Supplementary file 1 [file nutrients-15-00442-s001.zip › Questionnaire S1.pdf]

Questionnaire S1: NUTRITION HABITS OF PEOPLE ON A VEGETARIAN / VEGAN DIET DURING THE PANDEMIC

1. Place of residence*

2. Date of birth*

3. Sex*

- female
- male
- prefere not to say

4. Height [ft]*

5. Weight [lbs]*

6. Place of residence*

- countryside
- city with up to 50,000 residents
- city with up to 100,000 residents
- city with up to 250,000 residents
- city with over 250,000 residents

7. I consider myself*

- Vegetarian
- Vegan

8. For how many years have you been on a meatless diet?*

9. Current employment status*

- I'm a student
- I'm a student with a part time job
- I'm a student with a full time job
- I'm an office worker (ca.8h)
- I'm a manual worker (ca.8h)
- I'm not currently employed
- other:

10. What's your education?

- primary
- vocational
- secondary
- attending university
- higher in medical studies
- higher in biological studies
- higher in economical studies
- higher in mechanical studies
- other:

11. *only answer if you're a university student* What are you studying?

- Medical studies
- Biological studies
- Economical Studies
- Technical Studies
- other:

Questions about cardiovascular risk factors

1. During your last blood pressure measurement, your blood pressure value was as follows:

- > 140/80
- 140/80 - 120/60
- < 120/60
- I don’t know
- do not measure

2. How often do you eat meals prepared in deep fat?

- never/ almost never
- once a month or less often
- several times a month
- several times a week
- everyday
- several times a day

3. How often do you eat meals that include the addition of a large amount of fat (fast-food, pizza, ready meals)?

- never/ almost never
- once a month or less often
- several times a month
- several times a week
- everyday
- several times a day

4 How often do you add butter or cream (*or vegetable substitutes for these products) to your dishes?

- never/ almost never
- once a month or less often
- several times a month
- several times a week
- everyday
- several times a day

5 How often do you add vegetable oil (rapeseed oil, sunflower oil, olive oil, flaxseed oil, etc.) to your dishes?

- never/ almost never
- once a month or less often
- several times a month
- several times a week
- everyday
- several times a day

6. Please estimate how often you add to your dishes - 1 tablespoon of fat (oil / oil / butter / margarine / coconut oil / peanut butter):

- never/ almost never
- several times a month
- several times a week
- I consume < 1 tbsp per day
- I consume 1-2 tablespoons a day
- I consume 3-4 tablespoons a day
- I consume > 5 tbsp per day

7. do you smoke cigarettes?

- yes, occasionally
- yes, regularly
- no

8. do you consume alcohol?

- no
- once in a month or less
- several times a month
- approx. 3-5 times a week
- approx. 1-2 times a week
- everyday
- I refuse to answer

9. How do you determine your level of physical activity?

Level of physical activity	Number of minutes of moderate intensity exercise (e.g., dynamic walking or cycling, aerobics) performed
Low	<150 min/week
Medium	150–300 min weekly
High	>300 min/week

- low activity
- medium activity
- high activity

10. have/are there any cardiovascular diseases among first-degree relatives (parents, siblings, children), i.e. heart disease, heart attack, stroke, hypertension, thrombosis ?

- yes
- no
- don't know

11. did/does your first-degree relatives (parents, siblings, children) have abnormal lipid profile values (total cholesterol, triglycerides, HDL,LDL fraction cholesterol) ?

- yes
- no
- don't know

12. Did/does any first-degree relatives (parents, siblings, children) have abnormal fasting glucose values, or relatives have diabetes/insulin resistance?

- yes
- no
- don't know

13. Have you been diagnosed with cardiovascular diseases, i.e. heart disease, heart attack, stroke, hypertension, thrombosis ?

- yes (if YES, please write what irregularity the answer refers to - below in "other")
- no
- don't know
- other:……..

14. do you have abnormal lipid profile values (total cholesterol, triglycerides, HDL cholesterol, ,LDL cholesterol) ?

- yes
- no
- I don't know, I haven't had such research

15. if you answered yes to the previous question, please indicate which parameters are incorrect? (*Multiple answers can be marked)

- total cholesterol
- HDL cholesterol
- LDL cholesterol
- triglycerides
- don't remember

16. have you been diagnosed with abnormal fasting glucose values / diabetes / insulin resistance?

- yes (if YES, please write what irregularity the answer refers to - below in "other")
- no
- don't know
- other:……..

Changes caused by an epidemiological situation

1. Has your lifestyle changed due to the epidemiological situation?*

- yes
- no

2. Did the information on the number of cases and deaths from COVID have an impact on your well-being?*

- yes, negative
- no

3. What kind of feelings did the epidemic evoke? *you may choose more than one answer*

- fear, horror
- anger
- relief from having to isolate and maintain social distance
- hopelessness, helplessness
- calm

4. Did the information on Covid-19 have any impact on the number of meals consumed during the day?*

- yes, I was eating less
- yes, I was eating more
- no impact
- I have no opinion

5. Did your eating habits change during the pandemic? *You may choose more than one answer*

- yes, I was eating more healthy food, e.g. nuts, vegetables, fruit
- yes, I was eating less healthy food, e.g. nuts, vegetables, fruit
- yes, I was eating more sweets
- yes, I was eating less sweets
- yes, I was eating more savoury snacks
- yes, I was eating less savoury snacks
- yes, I was eating more junk food, e.g. ready meals, fast food
- yes, I was eating less junk food, e.g. ready meals, fast food
- no change

6. How did your activity routine change during the pandemic?*

- I exercised RARELY and the Covid-19 situation did not have any impact
- I exercised RARELY and the Covid-19 situation made me work out MORE
- I exercised RARELY and the Covid-19 situation made me work out even LESS
- I exercised SOMETIMES and the Covid-19 situation did not have any impact
- I exercised SOMETIMES and the Covid-19 situation made me work out MORE
- I exercised SOMETIMES and the Covid-19 situation made me work out LESS
- I exercised FREQUENTLY and the Covid-19 situation did not have any impact
- I exercised FREQUENTLY and the Covid-19 situation made me work out MORE
- I exercised FREQUENTLY and the Covid-19 situation made me work out LESS

7. Did your sleep pattern change during the pandemic?*

- yes, I slept longer
- yes, I slept shorter
- yes, I go to bed at more regular times
- yes, I go to bed at less regular Times
- I get up earlier
- I get up later
- I go to bed earlier
- I go to bed later
- no

8. How many hours of sleep did you get before the pandemic?*

- <5-6h
- 7-8h
- >8h

9. How many hours of sleep do you get during the pandemic?*

- <5-6h
- 7-8h
- >8h

10. Do you work more because of the Covid-19 situation?*

- yes
- no

11. What is the type of work you do during the pandemic period?

- I only work remotely
- I work hybrid (some days remote/some days on-site at work)
- I'm on the job every day (remote)
- not working during the pandemic period

12. Did your weight change during the pandemic?*

- I lost weight
- I gained weight
- no change
- I did not check

13. Did your eating habits change during the pandemic? *

- yes
- no (please, if you chose this then skip the next part and send the form)

Diet changes during the pandemic

1. During the pandemic, you consume every day:

- more food
- less food
- same amount of food as before the pandemic

2. Has the number of meals consumed during the day changed?

- yes
- no * (please go to question no. 5)
- don’t know

3. How many meals did you have before the pandemic?

- 1-2
- 3
- 4
- 5

- 6
- >6

4. How many meals per day did you have during the pandemic?

- 1-2
- 3
- 4
- 5
- 6
- >6

5. Has the regularity of eating meals changed?

- yes, during the pandemic I started eating more regularly
- yes, during the pandemic I started eating less regularly
- no

6. Did you start eating more snacks during the pandemic?

- definitely yes
- I consume more snacks
- I consume less snacks
- I'm consuming the same amount of snacks
- I did not consume snacks before and this not changed

7. Has food preparation changed during the pandemic?

- yes, I started to pay more attention to preparing a wholesome meal
- I have always prepared my own meals and nothing has changed in this regard
- nothing has changed, I consume ready/purchased meals as I did before the pandemic
- I started to consume more ready meals

8. During the pandemic, did you have ready meals more often?

- definitely yes
- yes
- no

9. Did you consume more vegetables during the pandemic?

- yes, I reach for them very often (compared to before the pandemic, I have increased my consumption by >3 pieces / day)
- yes, by about 1-2 pieces /day (compared to before the pandemic)
- no, vegetable consumption has not changed
- I don't like vegetables, I avoid eating them (also before the pandemic)

Please estimate the number of servings (vegetables) consumed during the day (assuming one serving is a glass of raw vegetables or half a glass of vegetable juice)

- <1 serving
- 1-2 servings
- <3 servings
- 4-6 servings

- 6-8 servings
- >8 servings
- don’t know

10. During the pandemic, did you consume fruit more often?

- yes, I reach for them very often (compared to before the pandemic, I have increased my consumption by >3 pieces / day)
- yes, by about 1-2 pieces /day (compared to before the pandemic)
- no, fruits consumption has not changed
- I don't like vegetables, I avoid eating them (also before the pandemic)

Please estimate the number of servings (fruit) consumed during the day (assuming that one serving is one average piece of fruit or half a glass of fruit juice)

- <1 serving
- 1-2 servings
- <3 servings
- 4-6 servings
- 6-8 servings
- >8 servings
- dont't know

11. During the pandemic, did you eat more legumes (chickpeas, lentils, beans, peas)?

- yes, I reach for them very often (compared to before the pandemic, I have increased my consumption by 3 times / day)
- yes, but I reach for them occasionally (1 time a day)
- no, consumption of legumes has not changed
- I don't like legumes, I avoid eating them (also before the pandemic)

Please estimate the number of servings (legumes) consumed during the day (assuming that one serving is half a cup of cooked legumes)

- <1 serving
- 1-2 servings
- <3 servings
- 4-6 servings
- 6-8 servings
- >8 servings
- don't know

12. During the pandemic, did you eat whole grains more often?

- yes, I reach for them very often (at least 5 times a day)
- yes, but I reach for them occasionally (<3 times a day)
- no, consumption of whole grain cereal products has not changed
- I don't like whole grain cereal products, I avoid eating them (including before the pandemic)

Please estimate the number of servings (whole grains) consumed during the day (assuming that one serving is one slice of bread)

- <1 serving

- 1-2 servings
- <3 servings
- 4-6 servings
- 6-8 servings
- >8 servings
- don't know

13. During the pandemic, did you eat protein products (plant substitutes) more often?

- yes, I reach for them very often (at least 5 times a day)
- yes, but I reach for them occasionally (<3 times a day)
- no, consumption of protein products has not changed
- I don't like protein products, I avoid eating them (including before the pandemic)

Please estimate the number of servings (plant protein products) consumed during the day (assuming that one serving is a glass of plant-based milk)

- <1 serving
- 1-2 servings
- <3 servings
- 4-6 servings
- 6-8 servings
- >8 servings
- don't know

14. During the pandemic, did you eat nuts, and seeds more often?

- yes, I reach for them very often (at least 5 times a day)
- yes, but I reach for them occasionally (<3 times a day)
- no, consumption of nuts and seeds has not changed
- I don't like this products, I avoid eating them (including before the pandemic)

Please estimate the number of servings (nuts, seeds) consumed during the day (assuming that one serving is 1/4 cup od walnuts)

- <1 serving
- 1-2 servings
- <3 servings
- 4-6 servings
- 6-8 servings
- >8 servings
- don’t know

15. Did you drink alcohol more often during the pandemic?

- yes, I reach for alcohol very often
- yes, but I reach for alcohol occasionally
- I consume the same amount of alcohol as before the pandemic
- I do not consume alcohol at all

16. Did you increase the number of cigarettes smoked during the pandemic?

- yes

- I started smoking during the pandemic
- I smoke the same amount during the pandemic
- I did not smoke before the pandemic and that did not change

17. I believe that the changes in my diet are:

- positive
- negative
- I have no opinion

18. What influenced you to change your diet (you can give more than 1 answer):

- stress, fear - negative emotions
- more time to prepare meals
- more time to consume snacks
- in this particular period, I wanted to take better care of my health
- other:
